# Non-invasive diagnosis of viability in seeds and lichens by infrared thermography under controlled environmental conditions

**DOI:** 10.1186/s13007-019-0531-8

**Published:** 2019-12-05

**Authors:** Beatriz Fernández-Marín, Othmar Buchner, Gerald Kastberger, Federica Piombino, José Ignacio García-Plazaola, Ilse Kranner

**Affiliations:** 1 Department of Botany and Center for Molecular Biosciences Innsbruck (CMBI), University of Innsbruck, 6020 Innsbruck, Austria; 2 Zoology Section, Institute of Biology, University of Graz, 8010 Graz, Austria; 3 Department of Plant Biology and Ecology, University of the Basque Country (UPV/EHU), Box. 644, 48080 Bilbao, Spain; 4 Department of Botany, Ecology and Plant Physiology, University of La Laguna, 38200 Tenerife, Spain

**Keywords:** Desiccation tolerance, Lichen, Thermal imaging, Seeds, Stress, Viability

## Abstract

**Background:**

Non-invasive procedures for the diagnosis of viability of plant or fungal tissues would be valuable for scientific, industrial and biomonitoring purposes. Previous studies showed that infrared thermography (IRT) enables non-invasive assessment of the viability of individual "orthodox" (i.e. desiccation tolerant) seeds upon water uptake. However, this method was not tested for rehydrating tissues of other desiccation tolerant life forms. Furthermore, evaporative cooling could obscure the effects of metabolic processes that contribute to heating and cooling, but its effects on the shape of the "thermal fingerprints" have not been explored. Here, we further adapted this method using a purpose-built chamber to control relative humidity (RH) and gaseous atmosphere. This enabled us to test (i) the influence of relative humidity on the thermal fingerprints during the imbibition of *Pisum sativum* (Garden pea) seeds, (ii) whether thermal fingerprints can be correlated with viability in lichens, and (iii) to assess the potential influence of aerobic metabolism on thermal fingerprints by controlling the oxygen concentration in the gaseous atmosphere around the samples. Finally, we developed a method to artificially "age" lichens and validated the IRT-based method to assess lichen viability in three lichen species.

**Results:**

Using either 30% or 100% RH during imbibition of pea seeds, we showed that "live" and "dead" seeds produced clearly discernible "thermal fingerprints", which significantly differed by > |0.15| °C in defined time windows, and that RH affected the shape of these thermal fingerprints. We demonstrated that IRT can also be used to assess the viability of the lichens *Lobaria pulmonaria*, *Pseudevernia furfuracea* and *Peltigera leucophlebia*. No clear relationship between aerobic metabolism and the shape of thermal fingerprints was found.

**Conclusions:**

Infrared thermography appears to be a promising method for the diagnosis of viability of desiccation-tolerant tissues at early stages of water uptake. For seeds, it is possible to diagnose viability within the first hours of rehydration, after which time they can still be re-dried and stored until further use. We envisage our work as a baseline study for the use of IR imaging techniques to investigate physiological heterogeneity of desiccation tolerant life forms such as lichens, which can be used for biomonitoring, and for sorting live and dead seeds, which is potentially useful for the seed trade.

## Background

Desiccation tolerant organisms are capable of surviving at water contents below 0.1 g water per g dry weight [1] and include life forms such as fungi, algae, bryophytes and some ferns, but relatively few angiosperms (the "resurrection angiosperms") and very few animals [2]. The so-called orthodox seeds, which are produced by an estimated 90% of seed plants, are also desiccation tolerant. Upon desiccation, including the drying process during seed maturation, the metabolism of desiccation tolerant organisms ceases, and restarts when water becomes available again. However, desiccation tolerant organisms cannot survive indeterminately, but suffer deteriorative processes that eventually culminate in viability loss. Therefore, they are attractive models to study the onset of metabolism upon water uptake. Tools that can diagnose how long desiccation tolerant organisms can remain in the desiccated state could be extremely valuable, especially for the seed industry, and for conservation projects aimed at wild plant seed conservation. Similarly, lichens are important bio-indicators of air quality, have high conservation value, and some are of economic value, for example to the perfume industry. Lichens are an intricate symbiosis between a fungus, termed "mycobiont", and one or more green algae or cyanobacteria, the "photobiont", and are also associated to a plethora of microorganisms [3]. It is believed that virtually all lichens are desiccation tolerant [4]. See [4–6] for further information on seed and lichen physiology, and viability loss and longevity in the dry state.

Non-invasive techniques allow analysing organisms without manipulation and direct contact, thereby minimising damage, injury or alteration of the process of interest. In the past two decades, different non-invasive imaging techniques such as chlorophyll fluorescence [7, 8], hyperspectral analysis [9] and infrared thermography (IRT) [10–13] have been increasingly used by plant scientists, for instance, to remotely monitor crop performance. Infrared thermography is a powerful non-invasive technique based on the measurement of infrared (IR) radiation emitted from an object, which is a function of surface temperature [13, 14] and emissivity [15]. Infrared cameras are capable of monitoring spatial distribution of temperature as well as thermal changes across surfaces over time. The high precision and relatively low cost of IRT have enabled a number of applications to be developed in plant and agricultural research [13].

The "energy balance" of a system comprises several components that determine the fluxes in and out of a system, which are reflected by its temperature [16]. A major component of this energy balance in plant tissues is evaporative cooling, and most IRT applications refer to physical and morphological processes driven, for instance, by water potential, transpiration or stomatal conductance [10, 13, 17]. Furthermore, IRT has been used for the detection of ice nucleation within tissues [18], heat accumulation in flowers [19], leaf cooling by thermal convection [20], photoprotective dissipation of energy absorbed by chlorophylls [21], and for the analysis of thermogenesis in the inflorescence of *Arum maculatum* or the spadix of *Symplocarpus foetidus* [22, 23], which in the latter is the result of cyanide-resistant respiration [24]. For comprehensive review of applications of IRT in plant biology see [15].

Infrared thermography has also been used to assess seed viability during imbibition [11]. Thermal imaging during the initial steps of orthodox seeds upon imbibition provided well-defined thermal signatures over time. These changes in seed surface temperature were related to biophysical and biochemical processes occurring in the imbibing seed tissues. An initial warming phase was related to the loss of kinetic energy as water bound to macromolecules such as starch. The subsequent rapid cooling was related to the dissolution of low molecular-weight carbohydrates such as glucose, maltose and raffinose, resulting in negative heat of solution, because energy is needed to dissolve the crystal structures of sugars that are present in dry seeds [11]. Seeds of different viability produced different thermal fingerprints that allowed predicting during the first 3 h of imbibition whether an individual seed will germinate or not [11]. This work [11] was pioneering in demonstrating the potential of IRT to study biophysical and biochemical processes related to the resumption of metabolic activity upon imbibition, and to develop a predictive tool for viability testing of orthodox seeds. Later, Kim and co-workers [25] used a different approach based on time-dependent thermal decay of lettuce seeds after artificial warming to evaluate seed viability. Furthermore, Men and co-workers [26] developed a new algorithm to diagnose seed viability by reproducing the method by Kranner et al. in 2010 [11] (see [27] for a recent review on non-invasive procedures for seed viability assessment).

Here, we aimed to further improve this method by paying special attention to evaporative cooling, which accompanies the imbibition of dry seeds and lichens. We constructed an incubation chamber, within which the relative humidity (RH) and also the gaseous atmosphere can be controlled, focussing on oxygen concentration. We first tested the influence of RH on the thermal fingerprints of imbibing pea seeds (*Pisum sativum* L.). Additionally, we tested whether thermal fingerprints can be correlated with viability in desiccation tolerant life forms other than seeds. We chose to study lichens, using mainly *Lobaria pulmonaria* as a model. For this lichen species, comprehensive background knowledge exists on the physiological responses to desiccation-rehydration cycles [28–32]. Furthermore, due to its sensitivity to air pollution, *L. pulmonaria* is widely used for biomonitoring studies [33–35]. We finally validated the IRT-based method to assess lichen viability for two further species, *Pseudevernia furfuracea* and *Peltigera leucophlebia*. To study lichen viability, we developed a method of "controlled deterioration" (CD), involving incubation of lichens at high RH (75%) and temperature (40 °C) as used in seed science and by the seed trade to age seeds artificially, to induce a decline in viability in a relatively short period of time. Unlike vascular plants, lichens lack stomata and cuticles, and under natural conditions are subjected to frequent changes in moisture content (MC), which are main drivers of thermal processes. Evaporative cooling during water uptake may potentially obscure the smaller signals related to biochemical or biophysical activity, reinforcing the need to assess the influence of evaporative cooling on the shape of their thermal fingerprints.

## Methods

### Seed and lichen material

Commercially available seeds of *Pisum sativum* L. cv Laxtons Progress No. 9 (Dehner, Innsbruck, Austria), with a percentage of 70% viable seeds, as indicated by the supplier, were used. Thalli of three lichen species were collected in the field. Thalli of *L. pulmonaria* (L. Hoffm.), a foliose lichen, were obtained from fallen trees in an oak forest at around 500 m a.s.l. in La Lastra, Northern Spain. The two other species were acquired in the vicinity of Innsbruck, Austria. *Pseudevernia furfuracea* (L.) Zopf, a fruticose species, was collected from *Picea abies* trees at 1950 m a.s.l. and *P. leucophlebia* (Nyl.) Gyelnik, a foliose species, was collected at 1720 m a.s.l from the ground of a *P. abies* woodland with *Vaccinium* spp. dominating the understorey. Only thalli with healthy appearance were used for the experiments. Thalli were air dried and then stored over silica gel at 4 °C in the dark until use. Before experimentation, thalli were preconditioned for 48 h in a growth chamber (Percival Intellus Environmental controller, CLF Plant Climatics, Emersacker, Germany) at 21 °C and 99.9% RH and a 12 h day/12 h night cycle with an irradiance of < 20 µmol photons m^−2^ s^−1^ (fluorescent light source PHILIPS, F17T8/TL841, Alto II™ Technology, USA) during the day period. After the preconditioning, thallus discs with a diameter (Ø) of 12 mm were excised with a cork borer from the foliose lichens *L. pulmonaria* and *P. leucophlebia*, whereas only small pieces of approximately 4 mm^2^ could be cut from the fruticose lichen *P. furfuracea*. Thallus discs or pieces, for simplicity termed "thallus discs" hereafter, were air dried and then kept above silica gel for 24 h before the experiments.

### Experimental design

**Fig. 1:**
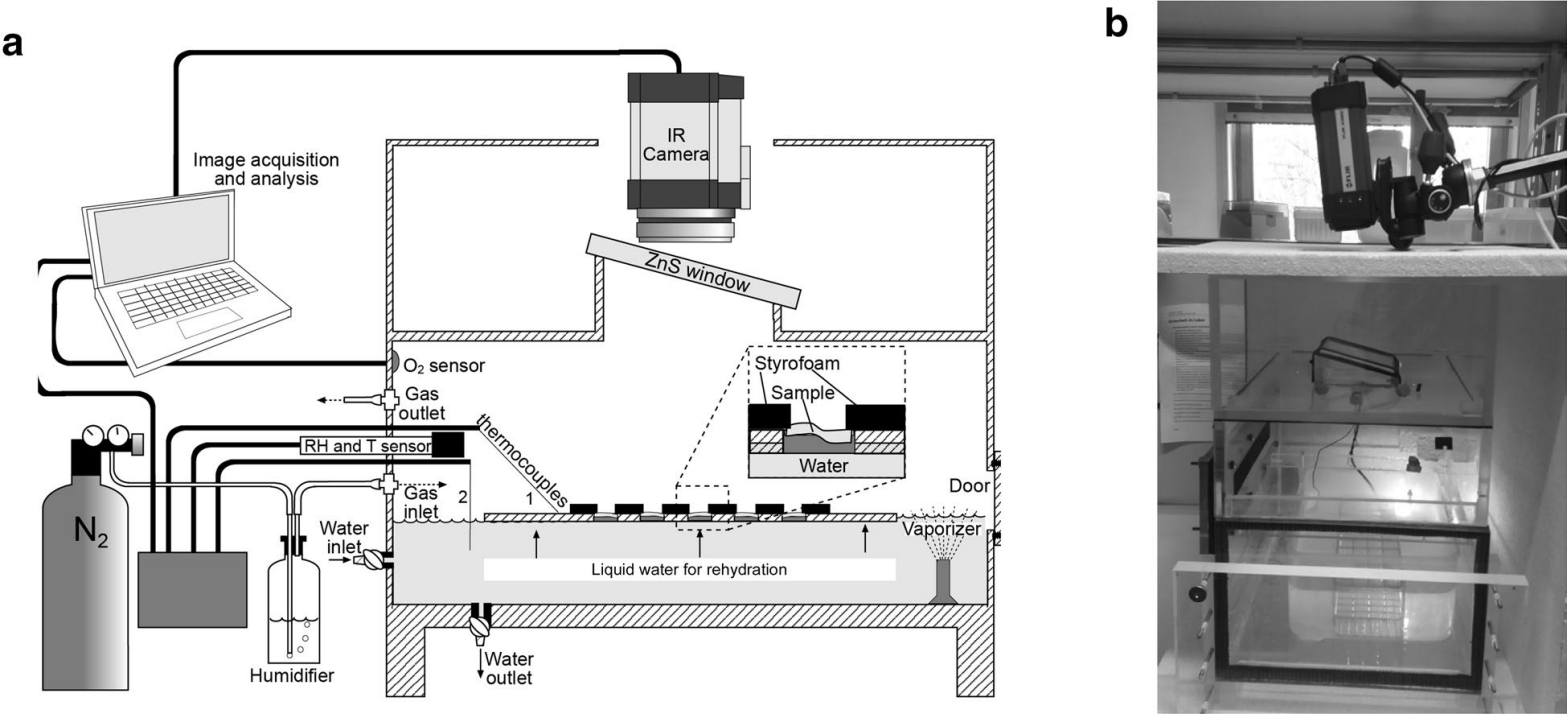
Design of a purpose-built chamber for IR analysis at controlled conditions of RH and gaseous atmosphere. **a** Schematic representation and **b** RGB image of the chamber. Thermal images were recorded with an IR-camera (FLIR A320) through an IR-transparent ZnS window, which was inclined at an angle of 15° to avoid reflexions. The environmental conditions of the atmosphere inside the chamber were monitored with a temperature and RH probe connected to a data logger, and O_2_ concentration was also measured (see "Methods" for specific conditions in each Experiment). Two thermocouples were used to monitor the surface temperature of the rack, and of the liquid water below. An ultrasonic water vaporizer was used to rapidly generate a RH > 98% upon rehydration in the chamber. The water table could be elevated until the filter papers were imbibed from below, directly providing the samples with liquid water. For experiments under anoxia, N_2_ gas was flushed through the chamber to replace the air, after humidifying the N_2_ by bubbling it through a bottle with deionised water. The dimensions of the chamber were 36 cm length, 30 cm width, 44 cm depth, and 36 × 30 × 30 cm for the bottom part containing the samples. See "Methods" and Additional file 1 for further details

A purpose-built incubation chamber was constructed (see Fig. 1 and Additional file 1 for detailed description of the chamber and of the control of environmental conditions inside of it) and used for five different experiments as detailed below (summarised in Table 1).

**Table 1:** Summary of treatments, materials and atmospheric conditions used for the different experiments

Experiment	Description	Type of material	Atmosphere	Sample specification
Exp. 1	Effects of relative humidity on the thermal fingerprints of *Pisum sativum* seeds	Seed (*P. sativum*)	Normoxia, 30% RH	"Live": germinated (*n* = *20*) “Dead”: non-germinated (*n* = *10*)
			Normoxia, 100% RH	"Live": germinated (*n* = *20*) “Dead”: non-germinated (*n* = *10*)
Exp. 2	Effects of imbibition with water vapour and liquid water, and oxygen, on the thermal fingerprints of *L. pulmonaria* thallus discs	Lichen (*L. pulmonaria*)	Normoxia, 100% RH	"Live": untreated (*n* = *15*) "Dead": microwaved (*n* = *15*)
			Anoxia, 100% RH	"Live": untreated (*n* = *15*) "Dead": microwaved (*n* = *15*)
Exp. 3	Thermal fingerprints of the fungal tissue in the lower cortex of *L. pulmonaria* discs	Lichen (*L. pulmonaria*) “upside down”	Normoxia, 100% RH	"Live": untreated (*n* = *14*) "Dead": microwaved (*n* = *14*)
Exp. 4	Effects of controlled deterioration on the thermal fingerprints of *L. pulmonaria*, *P. furfuracea* and *P. leucophlebia* thallus discs upon imbibition	Lichens (*L. pulmonaria, P. furfuracea* and *P. leucophlebia*)	Normoxia, 100% RH	"Live": untreated (*n* = *14*) "Stressed": 0.3 < Fv/Fm < 0.4 after CD (*n* = *14*) "Dead": Fv/Fm ≤ 0.2 after CD (*n* = *14*)
Exp. 5	Thermal fingerprints of low- and high-molecular-weight carbohydrates upon hydration	Carbohydrate standards	Normoxia, 100% RH	Starch (*n* = *8*) Glucose (*n* = *8*) Mannitol (*n* = *8*) Ribitol (*n* = *8*)

Numbers of replicates are shown in brackets

#### Experiment 1: Effects of relative humidity on the thermal fingerprints of *Pisum sativum* seeds

**Fig. 2:**
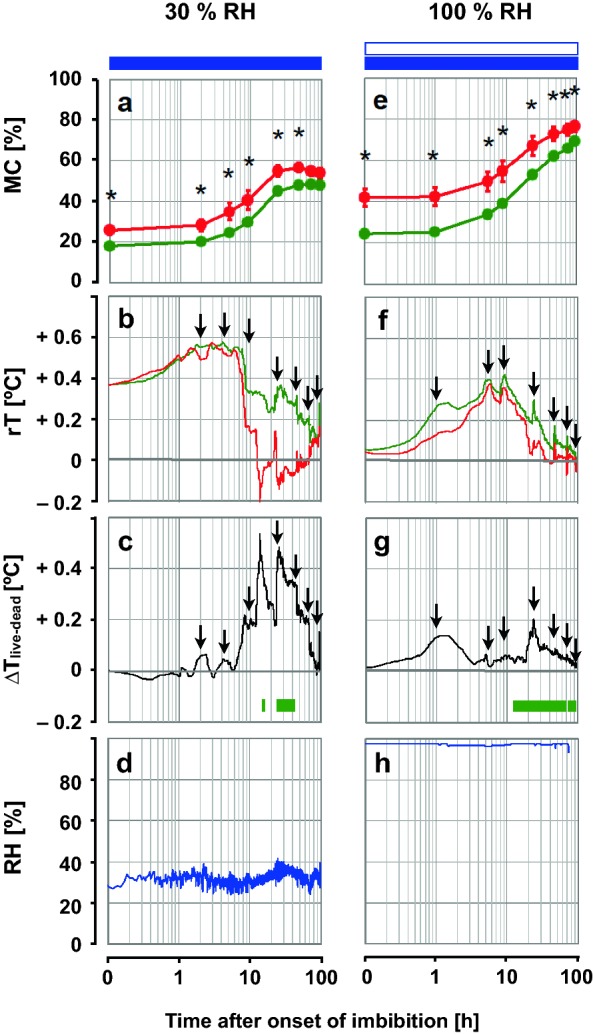
Effects of relative humidity on the thermal fingerprints of *Pisum sativum* seeds (Experiment 1). The left panels refer to imbibition of pea seeds at a target RH of 30% (termed "30% RH") and the right panels refer to imbibition at RH close to 100% ("100% RH"). In both cases, seeds were wetted from below with liquid water. **a**, **e** Seed moisture content (MC) of "live" (green line) seeds that germinated and "dead" (red line) seeds that did not germinate. Data show means ± SE (n = 10 "dead" seeds; n = 20 "live" seeds). Asterisks above the symbols denote significant differences in MC between "live" and "dead" seeds (*P* < 0.05; Mann–Whitney U test). **b**, **f** Thermal fingerprints of "live" and "dead" seeds, showing median values of relative temperature (rT) during imbibition. Arrows indicate interruptions between IR measurements during the weighing of seeds that were temporary taken out of the chamber for MC assessment (see “Methods”). **c**,** g** Differences between the fingerprints of "live" and "dead" seeds. Horizontal green bars indicate the time windows in which the T values of "live" seeds differed significantly from that of "dead" ones (*P* < 0.05; Two Sample t-test). **d**,** h** Relative humidity (RH) surrounding the seeds during the thermal recording. Open blue horizontal bars indicate the time periods of hydration by water vapour and closed blue bars indicate imbibition in liquid water from below

To test the influence of evaporative cooling on the thermal fingerprints of imbibing seeds, Experiment 1 was conducted to compare the thermal fingerprints of two sets of samples of 30 pea seeds each imbibed at "30% RH" and "100% RH", respectively. Imbibition of seeds at ≈ "30% RH" was conducted under ambient conditions outside the incubation chamber, with the rack on which the seeds were placed in contact with deionized water so that the seeds were wetted from below, without flooding them (RH values as assessed in the close environment of the samples throughout this Experiment 1 are shown in Fig. 2). IRT was recorded during the first 96 h of imbibition. At times 0, 1, 5.5, 9, 24, 47.5, 74.5 and 96 h after the onset of imbibition, the rack was briefly removed from below the IR camera and every individual seed was weighed for the assessment of MC. The time points of these interruptions are indicated by black arrows in Fig. 2c–f. This procedure adds noise to the thermal profiles, but allowed to pair the thermal fingerprints with the MC of individual seeds. Care was taken to place the pea seeds with their hilum facing downwards in direct contact with the damp filter paper. Once the IRT recording was finished, non-germinated seeds were incubated for 4 further days on wet "Seed Testing Paper" (Gd 3644 Blotter Blue, Whatman, UK) at 22 °C, 100% RH and a 12/12 h day/night cycle. This allowed us to assess the percentage of total germination (which was consistent with the percentage of total germination reported by the supplier), and to identify "live" and "dead" seeds. For simplicity, we refer to seeds as "live" and "dead" with reference to whether they germinated or not, respectively, after 8 days of incubation (please note that dormancy was bred out of garden pea, and therefore, lack of germination after 8 days—when the dead seeds started disintegrating and became mouldy—clearly indicated that the seeds were dead, not dormant). After germination, seeds were immediately dried at 103 °C for 24 h to estimate the dry weight (DW) of each individual seed. Seed viability ("live" or "dead") was recorded for each individual seed, so that thermal fingerprints could be assigned to each seed. Once the state of viability, "live" or "dead", was determined for each individual seed, "live" and "dead" seeds were grouped for further data evaluation.

#### Experiment 2: Effects of imbibition with water vapour and liquid water, and oxygen, on the thermal fingerprints of *L. pulmonaria* thallus discs

To test the suitability of the procedure for viability assessment of lichen samples, and to additionally consider the potential influence of aerobic metabolism on the thermal fingerprints, Experiment 2 was conducted with the lichen *L. pulmonaria* under normoxia (ambient oxygen) and anoxia (no oxygen).

Discs of different viability were generated: "Live" discs were left untreated, and represent high viability, which was also confirmed by their values of maximal photochemical efficiency of photosystem II (PSII) (Fv/Fm, see below). "Dead" discs represent the non-viable state with Fv/Fm ≤ 0.2. For Experiment 2, "dead" lichen samples were obtained by microwaving hydrated discs at 800 W for 30 s. This treatment led to a fast rise in thallus temperature ≥  60 °C that killed them, due to the sensitivity of lichens to heat shocks when hydrated [36]. "Live" (untreated) and "dead" (treated) discs were kept in the hydrated state at room temperature and at 7 µmol photons m^−2^ s^−1^ dim light (fluorescent lamp, PHILIPS, F17T8/TL841, Alto II™ Technology, USA), for 1 h, to allow all thallus discs to reach the same temperature before IR analysis. In contrast to pea seeds, which need more than a day to complete imbibition, lichen imbibe very fast, within minutes. Therefore, lichens were first exposed to water vapour for 10 min, which slows imbibition down, prior to exposure to liquid water.

"Live" and "dead" thallus discs were rehydrated under either normoxic and or anoxic conditions. For each treatment ("live" or "dead"), five replicate thallus discs and three repetitions of the experiment were performed. In the anoxia experiment, the thallus discs were placed in the chamber at an O_2_ concentration of 0%, which increased to approximately 0.1% after 1 h and up to a maximum of 0.6 ± 0.1% O_2_ by the end of the experiment. The Fv/Fm of each thallus was measured before and after the experiment to assess viability. A separate sample set was used to measure thallus MC at 0, 10, 15, 25, 40, 70, 100 and 160 min during rehydration (n = 5 thallus discs per time point and treatment). In this way, thallus MC was measured at intervals during rehydration, without the need to disturb the IR measurements, but only mean values of thallus MC and surface temperature can be compared.

#### Experiment 3: Thermal fingerprints of the fungal tissue in the lower cortex of *L. pulmonaria* discs

To test if the method described for Experiment 2 can also be used to assess the viability of fungal tissue, Experiment 3 was conducted under normoxia, using thallus discs of *L. pulmonaria* with the upper cortex, in which the algal layer is located, placed upside down, so that the lower cortex, consisting of fungal tissue, faced the IR camera*.* In this way, the surface temperature (T) of the mycobiont was studied. "Live" and "dead" thallus discs (n = 14) were used for the IR-recording. Fv/Fm was measured in each thallus disc after the experiment to additionally assess the viability of the photobiont. The same thallus discs were afterwards used for viability staining of the mycobiont (details below). For estimation of thallus viability, staining with 1% nitroblue tetrazolium (NBT; Sigma-Aldrich, Germany) was used to assess cell viability as a function of redox potential [37]. Actively respiring cells convert the water-soluble NBT to a dark blue precipitate. As for Experiment 2, a separate sample set (n = 14) was used to measure thallus MC at 0, 10, 15, 50, 100 and 170 min during rehydration.

#### Experiment 4: Effects of controlled deterioration on the thermal fingerprints of lichens

Experiment 4 was intended to test the applicability of IRT for the assessment of viability in desiccation tolerant life forms subjected to CD other than seeds. Thalli of *L. pulmonaria*, *P. furfuracea* and *P. leucophlebia* were exposed to a CD treatment of 75% RH and 40 °C in darkness. For each species three sets of samples with high, medium or low viability were obtained, referred to as "live", "stressed" or "dead" samples. This level of CD-related viability was estimated in n = 8 thalli per treatment and species by the value of maximal photochemical efficiency (Fv/Fm). The threshold level Fv/Fm ≥ 0.6 indicates good photochemical performance [30, 38–40] characteristic of highly viable, untreated "live" samples. Note that maximal Fv/Fm values for lichens are lower than those found in vascular plants [41]. "Stressed" samples with lowered photosynthetic performance (Fv/Fm 0.3–0.4) were obtained after 1.5, 2.5 or 4 days of CD for *P. furfuracea*, *L. pulmonaria* and *P. leucophlebia*, respectively. "Dead" samples (Fv/Fm ≤ 0.2) were obtained after 5.5, 6.5 or 9 days of CD for *P. furfuracea*, *L. pulmonaria* and *P. leucophlebia*, respectively. Three different runs (one per species: *L. pulmonaria, P. furfuracea, P. leucophlebia*) consisting of "live", "stressed" and "dead" thallus discs (n = 14 per treatment) were conducted. As in Experiments 2–3, a separate sample set was used for each species for the assessment of thallus MC at 0, 10, 15, 50, 100 and 170 min during rehydration (n = 4 thallus discs per time point and treatment).

#### Experiment 5: Thermal fingerprints of low- and high-molecular-weight carbohydrates upon hydration

To separately assess the thermal profiles of pure carbohydrates potentially contributing to the thermal profiles of biological samples [11], Experiment 5 was conducted using commercially available standards of carbohydrates. Soluble starch (Feinbiochemica, Heidelberg, Germany), d-(+)-glucose (Fluka, Steinheim, Germany), d-mannitol (Sigma-Aldrich, St. Louis, USA) and ribitol (Fluka, Steinheim, Germany) were studied, representative of carbohydrates usually accumulated by lichen photobionts or mycobionts. In Experiment 5, 20 mg of each carbohydrate was added to each empty well of Ø = 12 mm (n = 8) on the sample rack (described in Additional file 1: Methods and Figure S3. See also Fig. 1a), and the same procedure as in Experiment 4 was then followed for IR-recording.

### Chl *a* fluorescence measurements

Chlorophyll fluorescence, assessed by Fv/Fm, is widely used to estimate lichen photobiont viability (e.g. [36, 40]). For each thallus disc, Fv/Fm was determined with a chlorophyll fluorometer (Mini-PAM, Heinz Walz GmbH, Effeltrich, Germany) at the beginning and the end of each experiment. Thallus discs were fully hydrated and dark-adapted for at least 20 min to determine the minimum chlorophyll fluorescence yield (F_0_). Maximum chlorophyll fluorescence (Fm) was determined by a saturating pulse of 0.8 s and 6000 μmol photons m^−2^ s^−1^ [42]. This short pulse prior to IRT was performed approximately 20 s before the IR recording and no effect on the surface temperature of the thalli was observed. Variable chlorophyll fluorescence (Fv) was calculated as Fm–F_0_. The ratio Fv/Fm, which represents the maximum photochemical efficiency of PSII, was used to estimate the viability of the photobiont.

### Infrared thermography

Infrared images were recorded at a speed of 1 frame per minute during 4 days (seeds; Experiment 1) or 1 frame per second during 170 min (lichens, Experiments 2–4) with a FLIR A320 (FLIR, USA) camera, generating a data set of > 8000 frames per experiment. The IR camera was equipped with an uncooled microbolometer focal plane array detector, with a spectral range of 7.5–13 μm wavelength, an IR resolution of 320 × 240 pixels and a thermal sensitivity < 0.05 °C at 30 °C. Emissivity was set at 0.96 as is typically used for plant material [15], and RH recorded (values shown in Additional file 1: Table S1). Infrared images were taken at an approximate distance of 0.5 m above the samples and analysed in the img-format following a modification of the method described in [11]. Using the ResearchIR 4 software (FLIR Systems, Inc., USA), rectangular "regions of interest" (ROIs) with an area of 50–100 pixels were placed in the centre of each sample area and on several reference areas taken from filter paper covering empty wells of the sample rack (Additional file 1: Fig. S1). Each ROI covered approximately 90% of each sample surface, thus representing its overall surface temperature. The absolute temperature values (T) were averaged over all pixels per sensor area. The relative temperature (rT) of a sample at a certain time point t_i_ was calculated as rT (°C) = T_sample_ (t_i_) ‒ T_reference_ (t_i_) according to [11]. The temperatures at defined spots on the filter paper without seeds or lichen discs were used as references (T_reference_). This allowed considering sample-specific effects in temperature by subtracting the temperature of the filter paper on which the samples were laid. For each time point (t_i_), the difference in temperature between "live" (or "stressed") and "dead" samples was calculated as ΔT (°C) = T_live_ (t_i_) ‒ T_dead_ (t_i_). For Experiment 2, three repetitions were conducted, and the thermal profiles of the three runs pooled together. The time point at which liquid water reached the sample rack was roughly the same, but the fastest run was 66 s ahead of the slowest. Strong thermal effects occurring upon contact with liquid water were used for the synchronization of the data of the three runs. For that, in each run, the time at which the maximum (max) difference in temperature between two consecutive frames occurred (maxΔrT_*fii*-*fi*_/s (°C s^−1^); where *fi* represents a frame of the thermal recording, and *fii* the next frame) was taken to define the moment when the lichen disks came into contact with liquid water.

### Statistics

Fv/Fm or MC data were tested for significance before and after each experiment and between treatments using the Mann–Whitney U test (when comparison was needed between two groups of data) or the Kruskal–Wallis H test followed by the Kolmogorov–Smirnov test (for comparison of more than two groups of data). To test the significance of differences in rT between "live" and "dead" samples (in Experiments 1–3) and between "live" (or "stressed") and "dead" thallus discs (Experiment 4) along > 8000 time points per experiment, the two sample t-test was used (*P* < 0.05), and medians were taken to disregard outliers. The temperature range in which 99% of the camera noise occurred (ΔT_thr_ =  ± 0.05 °C, Additional file 1: Fig. S2) was determined to assess if the IR data in the same image differed with P < 0.05, i.e. if the differences between the median values of the thallus discs were bigger than their noise (|ΔT| >|ΔT_thr_|), which means that these differences occurred in a non-incidental manner and can therefore, be interpreted as biochemically or biophysically relevant thermal effects. Statistical analyses were performed with Microsoft^®^ Excel^®^ 2011 for Mac v14.4.7 and with IBM SPSS Statistics v24.

## Results

### Effects of relative humidity on the thermal profiles of pea seeds upon water uptake

In Experiment 1, MC and thermal fingerprints of "live" and "dead" pea seeds were monitored for 96 h after the onset of imbibition (Fig. 2). One set of seeds was imbibed at "30% RH" (Fig. 2a–d) and a second set of seeds was imbibed separately under "100% RH" to suppress evaporation (Fig. 2e–h). The RH values measured were very close to the target RHs of 30% and 100% (Fig. 2d, h; average, maximum and minimum values are shown in Additional file 1: Table S1).

Water uptake started within the first hours after the onset of imbibition. Under "100% RH", seeds reached higher maximum MCs than under "30% RH" (Fig. 2a, e). "Dead" seeds had higher MCs than "live" seeds at nearly all intervals of imbibition. In all seeds, rT (i.e., the difference between seed ROI and reference ROI) increased to above 0.4 °C within the first 10 h of imbibition and then dropped slowly and equilibrated with ambient temperature (rT = 0; Fig. 2b, f). Ten h after the onset of imbibition and thereafter, the temperatures of "live" and "dead" seeds differed during rehydration (Fig. 2c, g). For instance, at "30% RH", rTs of "live" pea seeds were significantly (at *P* < 0.05) higher than the rTs of "dead" seeds in the time intervals 15.9–17.4 h and 26.3–46.3 h (horizontal green bars in Fig. 2c). At "100% RH", the rTs of "live" seeds were significantly higher than those of "dead" seeds in the intervals 13.3–46.8 h, 48.6–70.5 h 71.0–93.9 h and 94.0–100 h (horizontal green bars in Fig. 2g). The rT values of both "live" and "dead" pea seeds (Fig. 2b, f) and ∆T_live–dead_ (Fig. 2c, g) were attenuated under "100% RH". The maximum value of ∆T_live–dead_ was 0.54 °C under "30% RH" atmosphere and 0.21 °C under "100% RH".

### Effects of anoxia on the thermal profiles of "life" and "dead" *Lobaria pulmonaria* thalli

**Fig. 3:**
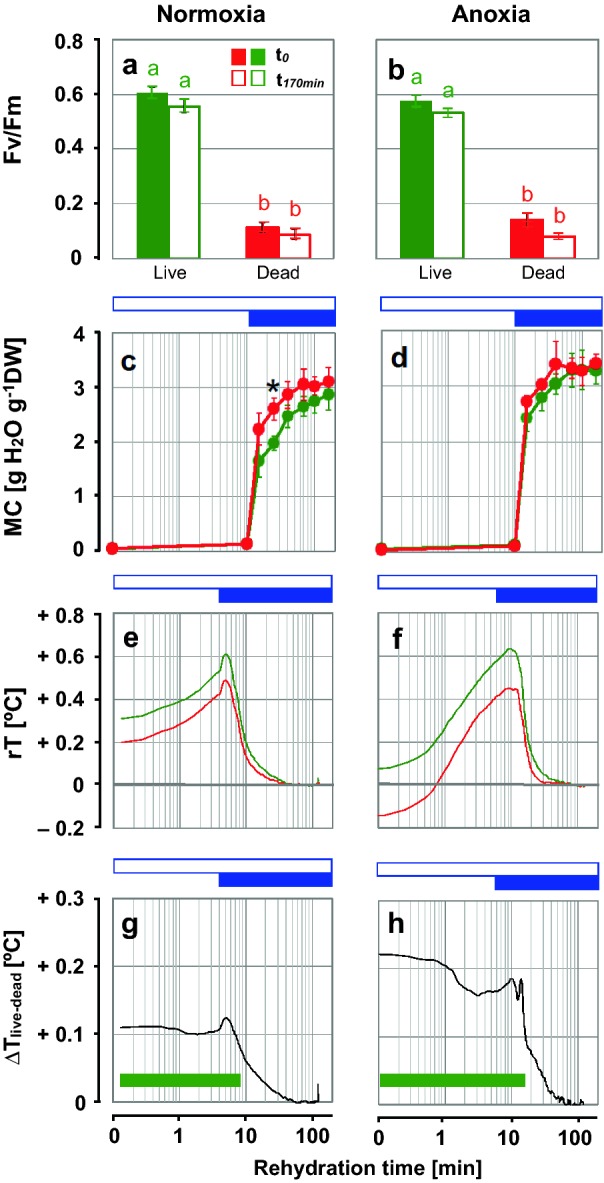
Effects of imbibition in water vapour and liquid water, and oxygen, on the thermal fingerprints of *L. pulmonaria* thallus discs (Experiment 2). Left panels show imbibition under normoxia (air) and right panels show imbibition under anoxia (air was replaced by flushing with N_2_ gas). "Live" samples (green) were untreated and "dead" samples (red) were killed by microwaving the hydrated thallus discs at 800 W for 30 s. **a**, **b** Viability of lichen discs before (t_*0*_; closed symbols) and after (t_*170min*_; open symbols) the experiment, assessed by the maximal photochemical efficiency of photosystem II. Data show means ± SE (n = 15 lichen discs). Green and red lower-case letters above the bars denote significant differences between treatments ("live", "dead") and between start (t_*0*_) and end (t_*170min*_) of the experiment (*P* < 0.05; Kruskal–Wallis test). **c**, **d** Moisture content (MC) of *L. pulmonaria* thallus discs during rehydration. Data show means ± SE of a second set of thallus discs (n = 5). The asterisk indicates that "life" and "dead" thallus discs differed significantly (*P* < 0.05; Mann–Whitney U test). **e**, **f** Thermal fingerprints of "live" and "dead" thallus discs showing median values of relative temperature (rT) during imbibition. Data are medians of n = 15 "live" and n = 15 "dead" thallus discs. **g**, **h** Differences between the fingerprints of "live" and "dead" thallus discs (ΔT_live–dead_). Horizontal green bars indicate the time windows in which the T values of "live" thallus discs differed significantly from that of "dead" ones (*P* < 0.05; Two Sample t-test). Open blue horizontal bars indicate the time periods of hydration by water vapour and closed blue bars indicate imbibition in liquid water from below

In Experiment 2, Fv/Fm values were highest in untreated, "live" samples, indicative of good viability, and decreased by ≈ 80% in "dead" samples (Fig. 3a, b). No significant differences were found between the Fv/Fm values measured before and after the infrared recording, indicating that the photosynthetic performance was not affected by the experimental conditions (Fig. 3a, b). "Live" thalli had comparable MCs than "dead" ones during most of the time course of rehydration (Fig. 3c, d). Significant differences were only found at the time point 25 min under normoxia (Fig. 3c).

During rehydration, rT of "live" and "dead" thallus discs were characterised by an increase above 0.4 °C within the first few minutes as thalli took up water vapour, followed by a further increase after direct contact with liquid water by up to 0.6 °C (Fig. 3e, f). After this peak, rT decreased progressively until equilibrium with ambient temperature. The rT values of "live" thallus discs were higher than that of "dead" thallus discs almost during the entire time course of rehydration (Fig. 3e, f). The maximum difference between "live" and "dead" (max ∆T_live–dead_) was 0.12 °C under normoxia (Fig. 3g) and 0.2 °C under anoxia (Fig. 3h). The ∆T_live–dead_ did not show any significant differences when normoxia and anoxia experiments were compared.

**Fig. 4:**
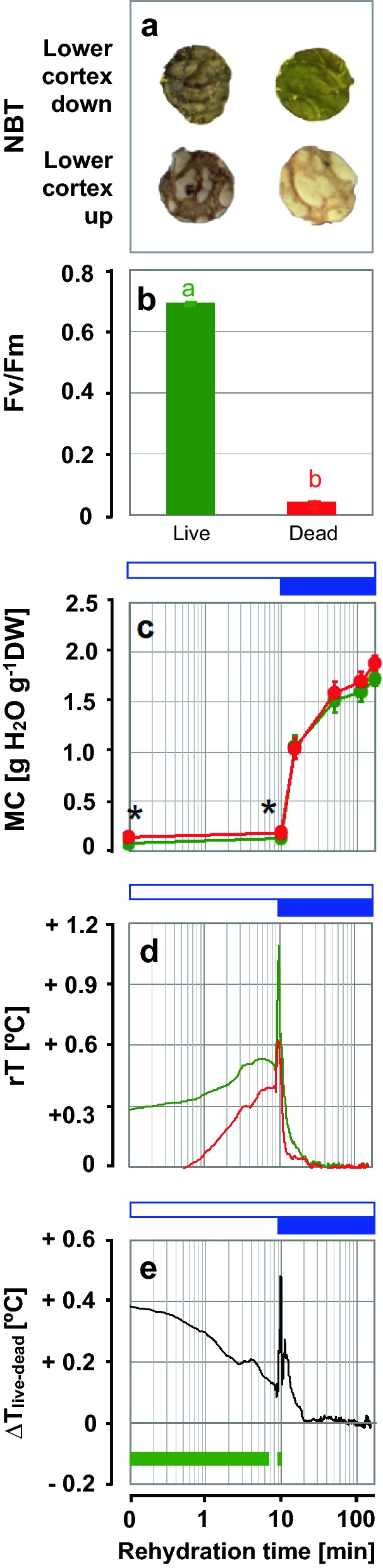
Thermal fingerprints of the fungal tissue in the lower cortex of *L. pulmonaria* discs (Experiment 3). Unlike in Fig. 3, the thallus discs were placed upside down to measure rT of the fungal layer. **a** Viability staining of "live" and "dead" thallus discs stained with 1% nitroblue tetrazolium (NBT). As actively respiring cells convert NBT to a dark precipitate, only "live" discs show dark staining. **b** Maximal photochemical efficiency (Fv/Fm) of "live" (green symbols) and "dead" (red symbols) thallus discs at the end of imbibition. Bars labelled with different letters indicate significant differences between treatments (*P* < 0.05; Kolmogorov–Smirnov test). Bars are means ± SE of (n = 14 lichen discs). **c** Moisture content (MC) of "live" (green) and "dead" (red) thallus discs during imbibition. Symbols are means ± SE (n = 14 discs). Asterisks indicate significant differences between treatments ("live", "dead") at *P* < 0.05 (Mann–Whitney U test) at each time-point. **d** Thermal fingerprints of "live" and "dead" thallus discs, showing median values of relative temperature (rT) during imbibition. Data are medians of n = 14 discs. **e** Differences between the fingerprints of "live" and "dead" thallus discs (ΔT_live–dead_). Green horizontal bars at the bottom of the panel indicate the time windows in which the rT values of "live" and "dead" thallus discs differed significantly (*P* < 0.05, Two Sample t-test). Open blue horizontal bars indicate the time periods of hydration by water vapour and closed blue bars indicate imbibition in liquid water from below

In Experiment 2, the lichen thalli were placed in their natural orientation, i.e. with the algae-containing upper cortex facing upwards (towards the IR camera), while the lower cortex was wetted by liquid water from below. In Experiment 3, the thallus discs were placed upside down, i.e. with the lower cortex facing the IR camera, so that thermal profiles of the fungal component were recorded (Fig. 4, see also Additional file 1: Fig. S4). Staining with NBT confirmed that "live" thallus discs had a high viability, and the lack of NBT staining on the lower cortex, where the photobiont is absent, showed that the treatment had killed the mycobiont (Fig. 4a); Fv/Fm measured at the end of the experiment showed very low photochemical efficiency of the photobiont of "dead" thallus discs (Fig. 4b). When exposed to water vapour, the MCs of "dead" thallus discs were higher (*P* < 0.05) than those of "live" ones, and after exposure to liquid water the differences became insignificant (Fig. 4c). Thermal profiles showed a characteristic time course with an initial increase during the exposure of samples to water vapour, followed by a sharp increase in temperature when samples were wetted with liquid water (Fig. 4d). Afterwards, the rT values converged to ambient temperature (rT = 0; Fig. 4d). The rT values of "live" thallus discs were higher than those of "dead" samples during the first 20 min of rehydration, with significant differences in the time intervals of 0–6.9 min and 8.8–9.7 min (*P* < 0.05) (Fig. 4e). In summary, the significant differences between "live" and "dead" *L. pulmonaria* thallus discs, obtained from the lower cortex in Experiment 3, confirmed that IRT can also be used to assess the viability of fungal tissue.

### Viability of lichen thalli of three different species after controlled deterioration

**Fig. 5:**
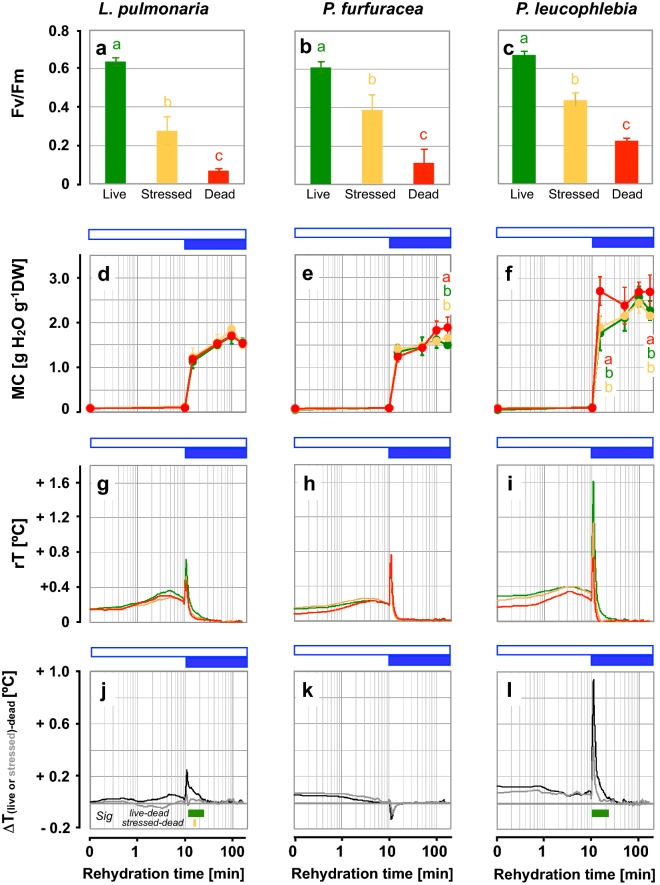
Effects of controlled deterioration on the thermal fingerprints of *L. pulmonaria*, *P. furfuracea* and *P. leucophlebia* thallus discs upon imbibition (Experiment 4). "Live", "stressed" and "dead" thallus discs are shown in green, orange and red colour, respectively. **a**–**c** Maximal photochemical efficiency (Fv/Fm) of thallus discs. Bars labelled with different letters indicate significant differences between "live", "stressed" and "dead" thallus discs with high, medium or low Fv/Fm, respectively, at *P* < 0.05 (one-way ANOVA). Bars are means ± SE of n = 8 thallus discs each. **d**–**f** Moisture contents (MC) of another set of thallus discs during imbibition. Symbols are means ± SE (n = 4). At each time interval, letters indicate significant differences between "live", "stressed" and "dead" thallus discs at *P* < 0.05 (one-way ANOVA). In d, the green and red lines overlap. **g**–**i** Thermal fingerprints of "live", "stressed" and "dead" thallus discs, showing median values of relative temperature (rT) during imbibition. Data are medians of n = 14 thallus discs. **j**–**l** Differences between the fingerprints of "live" vs "dead", and of "stressed" vs "dead" thalli (ΔT). Horizontal green (or orange) bars at the bottom of the graphs indicate the time windows in which the rT of "live" (or "stressed") discs differed significantly from "dead" discs at *P* < 0.05 (Two Sample t-test). Open blue horizontal bars indicate the time periods of hydration by water vapour and closed blue bars indicate imbibition in liquid water from below

The CD treatment of *L. pulmonaria*, *P. furfuracea*, and *P. leucophlebia* thalli produced sample sets with different viabilities (Fig. 5). The Fv/Fm values of "stressed" thallus discs decreased by ≈ 40% and of "dead" thallus discs by ≈ 75% compared to "live" thallus discs (Fig. 5a–c). "Live" thallus discs had comparable thallus MCs than "stressed" and "dead" ones during most of the time course of rehydration (Fig. 5d–f). Some differences in MC between live and dead samples were significant (*P* < 0.05) at timepoints t_170min_ after the onset of rehydration for *P. furfuracea* and t_15min_ and t_170min_ for *P. leucophlebia* (Fig. 5e, f).

Similar as observed in Experiments 2 and 3, thermal profiles during rehydration of thallus discs were characterised by a moderate initial increase in temperature under water vapour, followed by a sharp increase upon contact with liquid water from below (Fig. 5g–i). The thermal profiles obtained in Experiments 3 showed a much sharper increase in rT and a much sharper subsequent drop (Fig. 4d) compared to those obtained in Experiment 2 (Fig. 3e). These differences are caused by the experimental design. In Experiment 3, 14 discs per treatment ("life" and "dead") were run simultaneously. In Experiment 2, data from three repetitions, each with five thallus discs per treatment ("life" and "dead") were averaged, and the time periods when the water table reached the thalli differed slightly (by 66 s) between the three repetitions.

The magnitude of this rise in temperature varied among species and treatments. *P. leucophlebia* showed the biggest increase in rT, with a max rT of + 1.6 °C (Fig. 5i). This species also showed the largest ∆T when comparing "live" and "dead" samples (max ∆T_live–dead_ =  + 0.9 °C), or when comparing "stressed" and "dead" thallus discs (max ∆T_stressed-dead_ =  + 0.4 °C) (Fig. 5l). The rT values of "live" thallus discs were higher than those of "dead" ones during nearly the entire experiment for *L. pulmonaria* and *P. leucophlebia* and for the first minutes in *P. furfuracea*. Significant differences between "live" and "dead" *L. pulmonaria* thallus discs were found in the time interval 12.5–23.4 min (Fig. 5j) and for *P. leucophlebia* in the interval 9.9–22.2 min after the onset of rehydration (Fig. 5l). *Pseudevernia furfuracea* showed only small temperature differences between "live" and "dead" thallus discs (∆T_live–dead_ = 0.12 °C; Fig. 5h, k), which were below the threshold of the IR-camera noise and therefore not considered further. The minute differences found in *P. furfuracea* were consistent with the low biomass available from this fruticose lichen that could be mounted into the apertures of the rack (with an average size of 4 mm^2^ the thallus pieces were smaller by one magnitude compared to those of *L. pulmonaria* and *P. leucophlebia* with average disc sizes of 113 mm^2^).

**Fig. 6:**
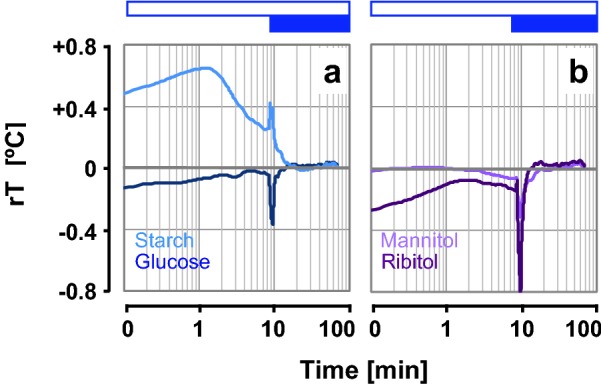
Thermal fingerprints of low- and high-molecular-weight carbohydrates upon hydration. **a** Thermal profiles of starch and glucose, both of which occur in seeds and lichens symbionts. **b** Thermal profiles of mannitol and ribitol, which frequently occur in lichens with green algal photobionts. Open blue horizontal bars indicate the time periods of hydration by water vapour and closed blue bars indicate imbibition with liquid water from below (as in Figs. 4, 5). Data are medians of n = 8 replicates

The carbohydrate standards of starch, glucose, mannitol and ribitol (Fig. 6) were subjected to the same hydration regime as lichen thalli in Experiment 4. When glucose, mannitol and ribitol were exposed to water vapour, the rT values were negative, indicating cooling. When starch was exposed to water vapour, the rT values were positive, corresponding to warming. Upon the subsequent contact with liquid water, the sugars and sugar alcohols showed a further strong cooling peak, and starch exhibited a strong warming peak, and after these peaks all substances equilibrated with ambient temperature (Fig. 6).

## Discussion

Imaging techniques are gaining importance for studying spatial and temporal patterns of metabolic activity or chemical composition in plants [7, 9–13] and are starting to be applied to lichens [43, 44], also offering the possibility to conduct large-scale surveys by analysing a high number of samples simultaneously, e.g. for selection of mutants or phenotype evaluation [10]. Based on previous experience with the assessment of viability in imbibing seeds using IRT [11], we further adapted this method to test the influence of evaporative cooling on thermal fingerprints during the imbibition of pea seeds, and to study the thermal profiles during rehydration in three lichens, *L. pulmonaria, P. furfuracea* and *P. leucophlebia*. We demonstrated that IRT enables non-invasive, simultaneous measurements of a number of replicate lichen samples, providing the basis for further studies into lichen physiology.

We first showed that our purpose-built incubation chamber was suitable for IRT analysis of imbibing pea seeds under constant low ("30%") or high ("100%") RH (Fig. 2). The low RH was chosen to allow evaporative cooling as in the paper by Kranner et al. 2010 [11], and "100% RH" was chosen to suppress evaporative cooling. At "30% RH", the thermal profiles of imbibing pea seeds shown here (Fig. 2b) were comparable with those shown earlier, albeit less pronounced [11]. The thermal profiles shown in Fig. 2 were not as smooth as those shown before [11], because seed samples were removed at several intervals in order to estimate the MC of each individual seed (arrows in Fig. 2b–g). However, the patterns of the thermal profiles of "live" and "dead" seeds shown here and earlier [11] share the following characteristics: rT showed (roughly) three phases, starting with an initial increase, followed by a sharp decrease and finally, equilibration with ambient temperature. Moreover, the sharp decrease in rT in "dead" seeds was much greater and sharper than in "live" seed (Fig. 2b and [11]). Importantly, the thermal profiles of "live" and "dead" seeds significantly differed from each other at certain time intervals (Fig. 2c and [11]).

Imbibition of pea seeds at "100% RH" also allowed distinguishing "live" from "dead" seeds (Fig. 2f, g), but only two phases were observed: rT was first dominated by warming and then fell again until equilibrium with ambient temperature, without a clear sharp drop after the initial warming. Therefore, "100% RH" suppresses evaporative cooling, but as a trade-off, the signal is dampened, compared to "30% RH". For diagnosing seed viability, lower RHs may be more useful, because the amplitude of rT in the thermal profile is greater (Fig. 2b, f) and the differences between "life" and "dead" seeds are more pronounced (Fig. 2c, g).

We then studied lichens to test if IRT can be used to non-invasively diagnose viability in rehydrating desiccation tolerant organisms other than orthodox seeds. As explained in "Methods", lichens were initially exposed to water vapour at "100% RH" for 10 min, and imbibition was rapidly completed when they came into contact with liquid water (Fig. 3e). As in seeds (Fig. 2f), the thermal profiles upon rehydration were first dominated by gradual warming upon exposure to water vapour, followed by a further rise in rT when thalli were wetted with liquid water, and then a decrease in rT and equilibration with ambient temperature. Overall, the thermal profiles of "live" lichen discs were warmer than those from "dead" discs (killed by microwaving) during rehydration (Fig. 3g, h). In summary, under "100% RH", the profiles of "live" and "dead" pea seeds and lichen discs were rather similar, dominated by warming in the first phase, followed by equilibration to ambient temperature. However, lichens underwent these changes at a much faster time scale (by an order of magnitude), and due to the two phases of imbibition using water vapour and then liquid water, a sharper peak was seen in lichens compared to seeds. As for seeds, the viability ("life" or "dead") of lichen disks could be assessed by IRT.

The initial warming upon imbibition was previously linked to the loss of kinetic energy, which is dissipated as heat, as water molecules bind to macromolecules such as cellulose, starch and proteins [11]. For comparison with earlier work [11], we observed the thermal profiles of low- and high-molecular-weight carbohydrates at the same experimental conditions used for lichens, i.e. imbibition by water vapour followed by liquid water. As do other desiccation tolerant organisms, lichens contain osmolytes that protect them from desiccation-induced injury [45], whose dissolution upon rehydration can contribute to cooling. Among the sugars and sugar alcohols that may produce negative heat of solution (i.e., cooling), mannitol was the most abundant low-molecular-weight carbohydrate found in various lichens, followed by ribitol in green algal lichens with *Trebouxia* sp. as photobionts, whereas cyanobacterial lichens also accumulate glucose [46]. Commercial standards of these low-molecular-weight carbohydrates (glucose, mannitol, ribitol) produced thermal fingerprints dominated by cooling, when subjected to the same hydration regime than lichen samples at "100% RH", with a sharp cooling peak upon contact with liquid water (Fig. 6). By contrast, the thermal profile of starch was dominated by initial warming, with a sharp warming peak upon contact with liquid water, followed by a subsequent cooling phase towards equilibrium with ambient temperature (Fig. 6a). Both, warming and cooling processes are likely to occur simultaneously during imbibition of seeds (Fig. 2) and hydration of lichens (Figs. 3, 4 and 5), but the first phase appears to be dominated by the interaction of water molecules with polymers such as starch and cellulose, and the second phase by cooling, related to the dissolution of low-molecular-weight carbohydrates as well as equilibration of samples with ambient temperature after all macromolecules were saturated with water.

Beckett et al. [47, 48] also observed that rehydration of lichens was accompanied by warming over a period of 4 h, measured by microcalorimetry. They also found a concomitant burst in respiratory activity [47] and suggested that the activation of mitochondrial alternative oxidase (AOX) or uncoupling proteins (UCP) could contribute to heat production. In flowers, AOX also contributes to thermogenesis to attract pollinators [49]. Furthermore, heat release by fungi has been related to fermentation [50], which in *Helleborus foetidus* flowers containing yeasts is able to raise nectar temperature by 6 °C [51]. Because AOX and/or UCP proteins could contribute to heat production, we investigated the contribution of aerobic metabolism to the thermal fingerprints by replacing the air in the chamber by N_2_. Figure 3b demonstrates that the gaseous atmosphere of N_2_ had no effect on the ability of the lichen thalli to recover Fv/Fm after the treatment. Unexpectedly, anoxic conditions did not diminish the thermal differences between "live" and "dead" thallus discs (Fig. 3e–h). Therefore, our results do not support the hypothesis that aerobic metabolism was a main factor responsible for the observed differences between “life” and “dead” thallus discs. Nevertheless, direct comparison between microcalorimetry [48] and IRT may not be appropriate. Infrared thermography allows measuring rehydrating samples immediately, whereas microcalorimetry requires that samples are left in the instrument for a short time before stable measurements can be achieved. Furthermore, in the present paper, rehydrating thallus pieces were permanently supplied with liquid water and water vapour (RH close to 100% at all times; Additional file 1: Table S1). In the work of Beckett et al. [48] wet lichen samples were placed in the microcalorimeter but were not supplied with water vapour and may have started losing water during the experiment. In the present work, heat flows were also strongly dominated by the rapid cooling of the filter papers on which the thallus discs were placed, and they were in direct contact with a large body of water. Taken together, these differences in experimental design and in the equipment used could explain why Beckett et al. [48] observed heat production for 4 h of rehydration, whereas in this work, the temperature of thallus discs equalled ambient temperature after 1 h already (Figs. 3, 4 and 5).

The two methods, microcalorimetry and IRT, may be useful for answering different questions, and the IRT method reported in this paper is intended to offer researchers further possibilities to study the metabolism of desiccation tolerant life forms. An advantage of IRT is that it can be used in open, but environmentally controlled systems close to natural conditions. We demonstrated that the biological materials used (seeds and lichen thalli) can be permanently exposed to water vapour during rehydration and can also be supplied with liquid water simultaneously, immediately or after chosen time points. The large water body with which the seeds or lichen discs were in contact, acted as a low-resistance medium that rapidly absorbed the heat generated by biophysical and biochemical processes, leading to rapid equilibration of the samples to the temperature of the filter papers on which the seeds or thallus discs were placed. This could be viewed as a disadvantage of the chamber system used here, because the large water body will have dampened the thermal fingerprints at both RHs. However, the most important point is that the thermal fingerprints of "live" and "dead" biological materials can be discerned: we demonstrated this (a) for "live" pea seeds with high viability compared to "dead" seeds that died without any experimental treatments (Fig. 2), (b) for "live" lichen thallus discs compared to "dead" ones killed by microwaving (Fig. 3) and (c) for "live" lichen thalli compared to "stressed" and "dead" ones after CD (Fig. 5). A faster dissolution of cellular molecules when "dead" thallus discs came into contact with liquid water, could be an explanation for their significantly lower rT values compared to "live" ones (Figs. 2, 3, 4 and 5). Although these differences could not be clearly related to thallus MC, at least in "dead" *P. leucophlebia* thallus discs, faster and greater water uptake was accompanied by a more pronounced fall in rT than in "live" ones (Fig. 5f, i and l). This agrees with earlier work reporting that heat treatments that kill lichens incur membrane damage [52], which could result in faster water uptake.

## Conclusions

The main aim of this work was to develop a method that enables IR imaging under controlled conditions of RH and gaseous atmosphere during rehydration, and a precise control of the rehydration method that can be applied to desiccation tolerant life forms such as orthodox seeds and lichens. Using IRT for investigating subtle differences in metabolic or non-metabolic processes is not trivial, because variations in temperature in biological systems result from intricately linked interactions of simultaneously occurring biophysical and biochemical processes. Different thermal profiles were found for "live" and "dead" seeds even under "100% RH" when evaporative cooling was supressed. For diagnosing seed viability, we recommend to use RHs below 100% to avoid dampening of the thermal signatures. This approach could be used for developing equipment for automated seed sorting, in combination with a robot that removes dead seeds. For desiccation tolerant cryptogams that rapidly take up water, such as lichens and bryophytes, slow rehydration under water vapour only is also an option, except for organisms that require liquid water to become metabolically active such as lichens with cyanobacterial photobionts [53, 54]. Finally, we also showed that CD, a method frequently used to age seeds, can be applied to lichens, which may be helpful for developing future protocols for a wider variety of desiccation tolerant organisms. In summary, our work contributes to advancing the use of IR imaging techniques for the study of spatial metabolic heterogeneity of micro-ecosystems such as lichens and soilcrusts, or economically important plant tissues such as seeds.

## Supplementary information

**Additional file 1.** Detailed description of the purpose-built incubation chamber and of the control of environmental conditions.

## Data Availability

The datasets used and/or analysed during the current study are available from the corresponding author on reasonable request.

## Abbreviations

a.s.l.: above sea level
AOX: alternative oxidase
CD: controlled deterioration
Chl: chlorophyll
DW: dry weight
ΔT: temperature difference
f: frame
Fv/Fm: maximum photochemical efficiency of photosystem II
IR: infrared
IRT: infrared thermography
max: maximum
MC: moisture content
min: minimum
NBT: nitroblue tetrazolium
ref: reference
PSII: photosystem II
RH: relative humidity
ROI: region of interest
rT: relative temperature
t: time
UCP: uncoupling proteins
