# Supplementary material for: Non-invasive diagnosis of viability in seeds and lichens by infrared thermography under controlled environmental conditions
Source: Plant Methods. 2019 Dec 5;15:147. doi: 10.1186/s13007-019-0531-8 (PMC6894116; doi:10.1186/s13007-019-0531-8)
Supplement: Supplementary file 1 — Additional file 1. Detailed description of the purpose-built incubation chamber and of the control of environmental conditions. [file 13007_2019_531_MOESM1_ESM.doc]

Additional file

**Additional file 1. Detailed description of the purpose-built incubation chamber and of the control of environmental conditions.:**

Incubation chamber

Plexiglas[®](http://dict.leo.org/ende/index_de.html#/search=®&searchLoc=0&resultOrder=basic&multiwordShowSingle=on) was used to build a chamber that enabled IRT measurements at controlled conditions of up to 99.8 % RH (Fig. 1). The IR camera was placed outside of the chamber to avoid heating of thallus discs by the radiation of the warm camera body. Images were taken through an IR-transparent ZnS "clear-grade glass" window (VITRON GmbH, Germany), which was inclined by 15° to avoid undesirable surface reflexions (Fig. 1). The chamber was divided into a top and bottom part, and the latter, with a volume of 30 L, contained the seed or lichen material. The temperature in the upper part was slightly higher due to the heat released by the IR camera, which sufficed to avoid condensation of water vapour on the ZnS window. A sealable door was built into one side of the chamber through which a rack with the seeds or thallus discs could be introduced into the chamber. Additionally, the chamber enabled control of the gaseous atmosphere, to produce either "normoxia" (ambient air with 21 % O_2_) or "anoxia" (0 % O_2_, achieved by flushing with N_2_ gas).

A sandwich-type rack was built for all experiments consisting of a plastic bottom part with apertures slightly larger than the biological materials (Ø =14 mm). Into these apertures, discs of filter paper (Ø =14 mm, 2 mm thickness, made of viscose) with an internal netting of organic cotton were placed (Natura Spontex sponge cloths, Spontex[®](http://dict.leo.org/ende/index_de.html#/search=®&searchLoc=0&resultOrder=basic&multiwordShowSingle=on)). Pea seeds (in Experiment 1) or lichen thallus discs (in Experiments 2, 3 and 4) were positioned on the filter papers, and a top layer of Styrofoam with apertures was clamped onto the bottom part. These apertures were slightly bigger than the dry peas for Experiment 1 (Ø = 14 mm) to allow seed expansion during imbibition. The apertures were slightly smaller than the lichen discs in Experiments 2-4 (i.e.: Ø =10 mm). This enabled the edges of the thallus discs to be pressed between the top and bottom layers of the rack, preventing curling of thallus discs during rehydration. The Styrofoam also served to insulate the individual (seed or lichen) samples from each other to avoid interference of thermal fingerprint between samples.

Control of environmental conditions

During each individual experiment, the room temperature was stable at 22 ± 1 °C. Imbibition of seeds and lichens was performed at dim light of 7 μmol photons m^-2^ s^-1^, which is below the light compensation point of *L. pulmonaria* [1], *P. furfuracea* [2] and *P. leucophlebia* [3]. All equipment and materials were equilibrated to ambient temperature for at least 24 h before the experiments.

For maximal water saturation of the air surrounding the biological materials, the bottom part of the incubation chamber was filled with approximately 3 L of de-ionized water. An ultrasonic vaporizer (Fogstar 100 L, Seliger, Villingen, Germany) submerged in the water at the bottom of the chamber allowed to rapidly generate high RH > 97 % (Fig. 1; Table S1). NB: technically, it is not possible to achieve 100 % RH; the measured values are shown in Table S1, but for simplicity, we refer to the maximal RH used as "100 % RH". The vaporizer was turned on for about 1 h to equilibrate the chamber, and then turned off. After inserting the seeds or lichen discs into the chamber, the vaporizer was turned on again for about 10 min. Any further use of the vaporizer was omitted to avoid heating of the chamber due to heat release from the instrument. Then, the water table was raised until it wetted the rack with the seed or lichen samples from underneath. When incubated at "100 % RH", imbibition of seeds or lichen discs started with the uptake of water vapour as soon as the rack was slid into the chamber. Then, the water table in the bottom part of the chamber was raised until it reached the filter paper, which took up water from below, allowing seeds or lichen discs to imbibe liquid water without being submerged.

For experiments under normoxia, the big volume of the chamber (30 L) in comparison with the small volume of seeds or lichen samples sufficed to maintain stable oxygen concentrations during the experiments (21 %). The O_2_ concentration inside the chamber was monitored with a minisensor oxygen meter (Fibox 3, PreSens, Nürnberg, Germany). To achieve anoxic conditions in Experiment 2, the chamber was flushed with N_2_ gas to replace all air before the measurements had started, and for another 10 min after inserting the rack with thallus discs. The gas outlet remained open during the replacement of air by N_2_ to avoid overpressure. The N_2_ gas was saturated with water by bubbling the "dry" N_2_ gas through a bottle with distilled water. This was necessary to maintain the high RH inside the chamber.

Temperature and RH data inside the chambers were monitored continuously with a temperature and RH probe (HMP45C, Campbell Scientific, Loughborough, UK) and recorded with a data logger (CR10, Campbell Scientific, Logan, US) in Experiments 1, 2, 3 and 5. The probe surface was protected by a breathable cover so that wetting of the surface by condensing water was effectively prevented.

**References cited**

1. Gaio-Oliveira G, Dahlman L, Máguas C, Palmqvist K. Growth in relation to microclimatic conditions and physiological characteristics of four *Lobaria pulmonaria* populations in two contrasting habitats. Ecography (Cop.). 2004;27:13–28.

2. Tretiach M, Crisafulli P, Pittao E, Rinino S, Roccotiello E, Modenesi P. Isidia ontogeny and its effect on the CO_2_ gas exchanges of the epiphytic lichen *Pseudevernia furfuracea* (L.) Zopf. Lichenol. 2005;37:445–62.

3. Solhaug KA, Xie L, Gauslaa Y. Unequal allocation of excitation energy between photosystem II and I reduces cyanolichen photosynthesis in blue light. Plant Cell Physiol. 2014;55:1404–14.

**Fig. S1** Example of false -coloured infrared thermographs of lichen thallus discs and filter papers used as references during rehydration under normoxia. Numerical values at the upper left corners indicate the time in minutes of rehydration at which the snapshots were recorded. White and black open squares, respectively, show the positions of the regions of interest (ROIs) within the "live" and "dead" thallus discs. White crosses mark the position of the digital sensors for the filter papers used as references. Panels **a** to **e** show successive changes at times 0.23, 8.12, 11.78, 12.97 and 25.03 minutes, respectively, after the onset of the rehydration. The colour scales at the bottom give the temperature ranges for desiccation and rehydration. The total length of the black and white scale bars represents 5 cm. (See also Fig. S4)

**Fig. S2** Evaluation of the camera noise. **a** Distribution of the changes in temperature between two consecutive frames (f_i_, f_i+1_) per s (ΔrT_fii-fi_ / s [°C · s^-1^]). The abscissa gives the values ΔrT_fii-fi_ / s (abbreviated further as ΔrT, see definition in Material & Methods), recorded for the sum of reference areas (n_ref_ = 14) in Experiment 2, referred to "desiccation under anoxia" (n_im_ = 8939 images). ΔrT values were sorted into 50 classes, whereby both data sets (ΔrT < 0: R^2^ = 0.9836; ΔrT > 0: R^2^ = 0.9779) were linearized in panel (a) displaying Gaussian distribution. Negative values represent cooling and positive values represent warming. Ordinate: probability of ΔrT values (P_ΔrT_) to occur in the range of ± 0.1°C s^-1^ (grey band). **b** Temporal distribution of ΔrT_fii-fi_ /s (abscissa) data over 170 min of measurement (ordinate) as summarized in panel **a**.

**Fig. S3** RGB images showing **a** a representative set of *Lobaria pulmonaria* thallus discs. (the wells cut into the Styrofoam had Ø = 10 mm) and **b** an example of one sample rack used below the Styrofoam. Thallus discs were clamped between two plastic plates with holes Ø = 10 mm. The holes in the bottom plate were equipped with spongy tissue which soaked up water, and the upper plate held the thallus discs in place so that they did not curl during water uptake.

**Fig. S4** RGB image showing a representative set of *L. pulmonaria* thallus discs after controlled deterioration and NBT staining. The upper panel shows the discs with the upper cortex upwards, and lower panel with the upper cortex downwards. L1 to L4 are live (untreated) samples. D1 to D5 are dead samples (microwaved at 800 W for 30 s in the hydrated state). Thallus discs are Ø = 12 mm. See Methods for details.

**Table S1.** Air relative humidity (RH), AirT, corresponding saturated vapour pressure (SVP) for the given average AirT and estimated Vapour Pressure Deficit (VPD), during hydration experiments monitored with a RH probe (HMP45C, Campbell Scientific, Loughborough, UK) and recorded with a data logger (CR10, Campbell Scientific, Logan, US) 5 cm above the samples. Absolute maximum (Max.), minimum (Min.) and average values are shown. These values were extracted from measurements taken every min during 96 h in Experiment 1, and from measurements taken every second during 170 min in all other Experiments.

|  |  |  | **RH [%]** |  |  | **AirT [°C]** |  | **SVP [KPa]** | **VPD [KPa]** |
| --- | --- | --- | --- | --- | --- | --- | --- | --- | --- |
| **Experiment** | **Description** | **Max.** | **Average** | **Min.** | **Max.** | **Average** | **Min.** | **Average** | **Average** |
| Exp. 1 | Pea seeds 30% RH | 41.6 | **32.6** | 24.1 | 22.6 | **21.1** | 22.1 | **2.503** | **1.687** |
|  | Pea seeds 100% RH | 97.8 | **97.4** | 93.3 | 25.4 | **24.6** | 23.6 | **3.094** | **0.080** |
| Exp. 2 | *L. pulmonaria* normoxia | 98.6 | **98.3** | 97.0 | 23.3 | **23.1** | 22.6 | **2.827** | **0.048** |
|  | *L. pulmonaria* anoxia | 99.2 | **99.0** | 98.4 | 22.9 | **22.7** | 22.1 | **2.760** | **0.028** |
| Exp. 3 | *L. pulmonaria* “upside down” | 99.8 | **99.2** | 98.9 | 23.2 | **22.9** | 22.4 | **2.793** | **0.022** |
| Exp. 5 | Standard carbohydrates | 99.7 | **99.0** | 98.5 | 21.8 | **21.5** | 21.1 | **2.565** | **0.026** |
